# Supplementary material for: Bullying Victimization Moderates the Association between Social Skills and Self-Esteem among Adolescents: A Cross-Sectional Study in International Schools
Source: Children (Basel). 2022 Oct 22;9(11):1606. doi: 10.3390/children9111606 (PMC9688646; doi:10.3390/children9111606)
Supplement: Supplementary file 1 [file children-09-01606-s001.zip › children-1917231-supplementary.pdf]

Prevalence of Bullying among Students in International Schools and Its Relationship with Social Skills and Mental Health Outcomes: A Study in Chiang Mai Province

Participant Code (Site/Number) ☐☐/☐☐☐

Eligibility Criteria

	Inclusion Criteria	Yes	No
1.	attending international schools in Chiang Mai province		
2.	aged from 13 to 18 years old		
3.	able to understand, read and write English		
	**Exclusion Criteria**		
1.	Students with blindness		

Demographic Data

What is your date of birth?

☐☐/☐☐/20☐☐

D D / M M / YYYY

What is your height (centimeter)?

☐☐☐.☐

What is your weight (Kilogram)?

☐☐☐.☐

What grade are you studying?

☐7 ☐8 ☐9 ☐10 ☐11 ☐12

What gender do you identify as?

☐ Female

☐ Male

☐ Other

What is your biological sex?

☐ Female

☐ Male

What is your ethnicity? (you can choose more than one)

☐ Asian (specify)_____

☐ American

☐ African

☐ European (specify)_____

☐ Other/ unknown

Which languages are you capable of speaking fluently? (Check all that apply)

☐ English

☐ Thai

☐ Chinese

☐ Japanese

☐ Korean

☐ Spanish

☐ Other (please specify)_____

Where were you born?

☐ Thailand

☐ China

☐ Japan

☐ Korea

☐ America

☐ Africa (please specify)_____

☐ Europe (please specify)_____

☐ Other (please specify)_____

Are you a day student or boarding student?

☐ Day

☐ Boarding

☐ Other (specify)_____

How long have you stayed in Thailand?

☐☐ year(s)

Who do you live with?

☐ On your own

☐ Friend(s)

☐ Parents

☐ Father

☐ Mother

☐ Other (please specify)______

If applicable, please specify your religion.

☐ Buddhism

☐ Christianity

☐ Catholicism

☐ Islam

☐ Hinduism

☐ Other (please specify)______

Do you have physical illness?

☐ Yes (please specify)______

☐ No

Do you have mental problems??

☐ Yes (please specify)______

☐ No

Have you ever used substance?

☐ Alcohol

☐ Smoking

☐ Cannabis

☐ Other (please specify)______

☐ None

How do perceive about your academic performance?

☐ Excellent

☐ Good

☐ Poor

☐ So so

☐ Bad

☐ Other

How much is your daily pocket money that you are given?

☐☐☐☐ THB

How many good friends in school?

☐ 0

☐ 1-2

☐ 3-7

☐ 8 and more

Do you have friends who you can trust? How many?

☐ 0

☐ 1-2

☐ 3-7

☐ 8 and more

In general, you can be yourself among classmates?

☐ Very much.

☐ Some.

☐ A little.

☐ Not at all.

How much you can be yourself among your best friends?

☐ Very much.

☐ Some.

☐ A little.

☐ Not at all.

MEASUREMENTS ITEMS

1. The OLWEUS BYLLY/VICTIM QUESTIONNAIRE (OBVQ)

1	2	3	4	5	6	7	8	9	10

11	12	13	14	15	16	17	18	19	20

21	22	23	24	25	26	27	28	29	30

31	32	33	34	35	36	37	38	39	40

2. Rosenberg Self-Esteem Scale (RSES)

1	2	3	4	5	6	7	8	9	10

3. Outcome Inventory-21 (OI-21)

1	2	3	4	5	6	7	8	9	10

11	12	13	14	15	16	17	18	19	20

21

4. Social skills Rating Scale (SSRS)

1	2	3	4	5	6	7	8	9	10

11	12	13	14	15	16	17	18	19	20

21	22	23	24	25	26	27	28	29	30

5. Resilient Inventory (RI-9)

1	2	3	4	5	6	7	8	9

6. Body Shape Questionnaire (BSQ)

1	2	3	4	5	6	7	8

7. Adolescent Discrimination Distress Index (ADDI)

1	2	3	4	5	6	7	8	9	10

11	12	13	14	15
